# *CD46* Genetic Variability and HIV-1 Infection Susceptibility

**DOI:** 10.3390/cells10113094

**Published:** 2021-11-09

**Authors:** Carmen Serrano-Rísquez, Mohamed Omar, María Amparo Gómez-Vidal, Luis Miguel Real, Juan Antonio Pineda, Antonio Rivero, Antonio Rivero-Juárez, Donald Forthal, Francisco J. Márquez, Sergio Lo Caputo, Mario Clerici, Mara Biasin, Antonio Caruz

**Affiliations:** 1 Unidad de Inmunogenética, Genética, Departamento de Biología Experimental, Universidad de Jaén, 23071 Jaén, Spain; csr00041@red.ujaen.es (C.S.-R.); jmarquez@ujaen.es (F.J.M.); 2 Servicio de Enfermedades Infecciosas y Microbiología Clínica, Complejo Hospitalario de Jaén, 23007 Jaén, Spain; omarampa@gmail.com (M.O.); ampagovi@gmail.com (M.A.G.-V.); 3 Servicio de Enfermedades Infecciosas y Microbiología Clínica, Hospital Universitario de Valme, 41014 Sevilla, Spain; lmreal67b@gmail.com (L.M.R.); japineda@us.es (J.A.P.); 4 Instituto Maimónides de Investigación Biomédica de Córdoba (IMIBIC), Hospital Universitario Reina Sofía, 14004 Cordoba, Spain; ariveror@gmail.com (A.R.); arjvet@gmail.com (A.R.-J.); 5 Division of Infectious Diseases, Department of Medicine, University of California, Irvine School of Medicine, Irvine, CA 92697, USA; dnfortha@hs.uci.edu; 6 Unit of Infectious Diseases, Department of Clinical and Experimental Medicine, University of Foggia, 71122 Foggia, Italy; sergio.locaputo@unifg.it; 7 Dipartimento di Fisiopatologia Medico-Chirurgica e dei Trapianti, University of Milan, 20122 Milan, Italy; mario.clerici@unimi.it; 8 Don C. Gnocchi Foundation, IRCCS, 20148 Milan, Italy; 9 Dipartimento di Scienze Biomediche e Cliniche “L. Sacco”, University of Milan, 20157 Milan, Italy; mara.biasin@unimi.it

**Keywords:** HIV-1, HIV exposed seronegative, HESN, *CD46*, complement

## Abstract

*CD46* is the main receptor for complement protein C3 and plays an important role in adaptive immune responses. *CD46* genetic variants are associated with susceptibility to several infectious and autoimmune diseases. Additionally, *CD46* function can be subverted by HIV-1 to evade attack by complement, a strategy shared by viruses of other families. We sought to determine the association between *CD46* gene variants and HIV-1 acquired through intravenous drug use (IDU) and sexual routes (*n* = 823). Study subjects were of European ancestry and were HIV-1 infected (*n* = 438) or exposed but seronegative (*n* = 387). Genotyping of the rs2796265 SNP located in the *CD46* gene region was done by allele-specific real-time PCR. A meta-analysis merging IDU and sexual cohorts indicates that the minor genotype (CC) was associated with increased resistance to HIV-1 infection OR = 0.2, 95% CI (0.07–0.61), *p* = 0.004. The HIV-1-protective genotype is correlated with reduced *CD46* expression and alterations in the ratio of *CD46* mRNA splicing isoforms.

## 1. Introduction

The complement system is a fundamental part of the innate and adaptive immune system and is required for the elimination of pathogens, immune complexes, and apoptotic cells. After exposure to pathogens, the core complement protein C3 is cleaved to C3b via the classical-lectin or the alternative pathways giving rise to the formation of active C3b convertases that trigger a massive conversion of C3b onto the adjacent pathogen surfaces [1]. The process continues until the formation of the membrane attack complex that destroys microbes through a pore in their membrane. In parallel, proteolysis of C3b to iC3b generates an opsonin that tags microbes for recognition and phagocytosis mediated by complement receptors expressed on phagocytes and lymphocytes [1,2]. This is a highly regulated process to avoid complement-mediated tissue injury. *CD46* (membrane cofactor protein [MCP]) belongs to the family of membrane-bound regulators of complement activation that inhibit off-target complement deposition and activation on host-cell surfaces [3]. *CD46* acts by preventing the formation of the C4b2a C3 convertase from the classical-lectin pathway or interferes with the conversion of factor B to Bb by factor D, thereby preventing the formation of the C3-convertase of the alternative pathway [1,2]. Other complement regulators are cofactors for complement factor I-mediated proteolysis and inactivation of C4b and C3b. This activity is assisted by a set of other soluble inhibitors of complement activation present in the serum, such as CFH or C4BP.

HIV can directly activate the classical cascade of the complement system, even in the absence of HIV-specific antibodies [4]. After seroconversion, the presence of antibodies further enhances complement deposition on the viral surface [5,6]. Furthermore, mannose-binding lectin, the trigger of the lectin pathway, can activate this branch of the complement system through binding to the HIV envelope [7]. Although high amounts of complement C3b are deposited on HIV viral surface, further complement-mediated lysis of the virus is quite inefficient [8]. The cause is that the virion membrane contains regulators of complement activation as *CD46* [9], that block the complement-dependent virolysis [10]. The use of anti-*CD46* antibodies renders HIV-1 susceptible to complement lysis giving rise to a significant reduction in HIV-1 infectivity [9].

Other viruses also incorporate *CD46* in the virion particles during the budding process, giving the pathogens protection against complement-mediated destruction. Human *CD46* or viral-encoded homologs are packaged as functional complement regulators in the outer membrane of several viral families such as the paramyxoviruses [11,12], rhabdoviruses [13], retroviruses [9,10], herpesviruses, and poxviruses [14].

*CD46* shows moonlighting properties [15] with additional and independent functions in several physiological processes including the initial phases of oocyte fertilization [16], neurogenesis, neuronal migration, synaptic remodeling [17]. Furthermore, *CD46* is an alternative receptor for the protein Jagged1 of the Notch signaling pathway [18]. The *CD46*-Jagged1 axis influences T lymphocyte proliferation, survival, and differentiation into a Th1 effector phenotype. Individuals with *CD46* or Jagged1 mutations do not develop Th1 responses and are extremely susceptible to infectious diseases during childhood [18]. *CD46* activation triggers intracellular signaling cascades in macrophages and dendritic cells giving rise to upregulation of pro-inflammatory cytokines such as IL12 or IFNα/β [19,20,21]. *CD46* also influences the migratory ability and cell-to-cell interaction of T lymphocytes and MHC-II antigen presentation [22]. Recently, a new autocrine *CD46*-mediated signaling has been discovered in T lymphocytes. That is based on the proteolysis of cytoplasmic-stored C3 present in lymphocytes. This proteolysis renders functionally relevant products (C3a and C3b) that are essential for T lymphocyte survival and proliferation [23,24].

*CD46* plays a key role in several infectious diseases and has been designated as a “pathogen magnet”. Unrelated microbes use this protein as the cell entry receptor. Several clinically relevant bacterial pathogens have evolved to adopt *CD46* as a receptor or attachment protein, including Streptococcus pyogenes [25], *Neisseria meningitidis*, *Neisseria gonorrheae* [26], *Escherichia coli* [27], *Klebsiella pneumonia* [28], and *Fusobacterium nucleatum* [29]. Measles virus [30], human herpesvirus 6 virus [31], cytomegalovirus [32], and adenoviruses of several serotypes [33] also utilize *CD46* as a receptor. The use of this common receptor goes beyond an easily available cell attachment protein. Rather, it is most likely the intracellular signaling cascades induced by *CD46* engagement that provide the pathogens with an advantage in the host, either by facilitating the rupture of protective host barriers or by creating a more permissive immune environment [34].

A puzzling observation is the alteration of the *CD46* mRNA splicing in HIV-1 infected cells, giving rise to a protein lacking an extracellular glycosylation domain [35]. The functional significance of this finding is not established, neither the differential physiological tasks of the *CD46* isoforms [35]. Additionally, significantly lower expression of *CD46* on the surface of dendritic cells [36], lung alveolar CD4+ lymphocytes [37], and peripheral blood mononuclear cells [38] of HIV-1 infected individuals compared to healthy donors were found, suggesting that modulation of *CD46* expression may influence HIV-1 infection and disease progression [36].

HIV-1 exposed seronegative (HESN) individuals are a heterogeneous group who remain HIV-1 uninfected despite exposure to the virus (intravenous drug and chemsex users, serodiscordant couples, men who have sex with men, and children born to HIV-1 infected mothers). Why infection after exposure appears to be resisted by some is likely due to multiple factors [39]. A better understanding of these factors may lead to novel prevention strategies, the discovery of antiviral targets, and the development of new vaccination strategies. In a previous study, using hypothesis-driven candidate gene strategies, we tested several hundred polymorphisms of complement-related genes and found one SNP located at the CR2 gene that was associated with HESN status in intravenous drug users (IDU) [40]. This finding was replicated in an independent population at risk of infection through sexual exposure. CR2 was later associated with the rate of infection after HIV-1 vaccination with rgp120 protein [41]. One SNP in the *CD46* gene was initially discarded due to a non-significant association with HIV-1 status after adjustment for multiple testing. This SNP showed a strong deviation from Hardy–Weinberg equilibrium. However, after a detailed analysis of the data, we found that the origin of this deviation was restricted to the HIV-1-infected individuals with one of the homozygous genotypes virtually absent in this population, suggesting a protective role during infection. In this current work, we have tested the potential role of this SNP as an HIV-1 resistance factor in independent populations (from Spain, Italy, and the USA) at risk of infection through sexual routes and performed a meta-analysis with the previous data obtained from IDU cohorts.

## 2. Materials and Methods

### 2.1. Study Population

We analyzed 823 individuals exposed to HIV-1 by the intravenous (*n* = 441) and sexual routes (*n* = 382) from Spain, Italy, and the United States. Among them, 438 HIV-1 were seropositive and 387 were considered as HESN. This group includes intravenous drug users, heterosexual discordant couples who routinely had unprotected sexual intercourse, and men who have sex with men (MSM). The MSM group includes Spanish individuals with high-risk behavior (chemsex) and placebo recipients participating in the Vax004 trial (Clinical-Trials.gov Identifier: NCT00002441) [42]. Samples are from available volunteers that became infected with HIV during the trial and a random sample of volunteers that remained uninfected. The main epidemiological and clinical characteristics of these volunteers have been previously described [41,42,43]. The present study included uninfected volunteers classified as having a high baseline risk of infection based on self-reported behaviors during the 6 months before enrollment. These behaviors were highly predictive of HIV infection in men [42]. Behavioral risk scores were calculated on the basis of the number of risk factors reported: unprotected receptive anal sex with an HIV-1-infected male partner; unprotected insertive anal sex with an HIV-1-infected male partner; unprotected receptive anal sex with an HIV-1-uninfected male partner; five or less acts of unprotected receptive anal sex with a male partner of unknown HIV-1 status; 10 or less sex partners; anal herpes; hepatitis A; use of poppers; and use of amphetamines. The inclusion criteria and main characteristics of these patients have been previously described in detail elsewhere [41,42,43].

### 2.2. Genotyping

DNA was extracted from frozen whole blood samples or saliva using the Quick Pure Blood DNA extraction kit. The *CD46* polymorphism was genotyped by allele-specific real-time PCR using the Biorad CFX equipment (Bio-Rad Laboratories Inc., Hercules, CA, USA). 

### 2.3. Biostatistics and Bioinformatics Analysis

Hardy–Weinberg equilibrium and genetic associations were assessed using the online tool (available online at https://ihg.gsf.de/cgi-bin/hw/hwa1.pl (accessed on 13 June 2021)) and the PLINK software (http://zzz.bwh.harvard.edu/plink/ (accessed on 12 June 2021)). Results from the sexual cohorts were combined with results from the IDU cohort using a fixed-effect meta-analysis. This meta-analysis was estimated with the online tool MetaGenyo developed at the “Centro Pfizer–Universidad de Granada–Junta de Andalucía de Genómica e Investigación Oncológica” (https://metagenyo.genyo.es/ (accessed on 13 June 2021)). This tool was also used for odds ratios (ORs) representation in Forest plots.

Levels of mRNA expression according to *CD46* rs2796265 genotypes were calculated using the GTEx portal (https://gtexportal.org/home/snp/rs2796265 (accessed on 19 June 2021)) Analysis Release V8 (dbGaP Accession phs000424.v8.p2) [13]. This portal allows testing of associations between SNPs and expression quantitative trait loci (eQTL) and splicing quantitative trait loci (sQTL) [44].

### 2.4. Ethics

This study was designed and performed according to the principles of the Helsinki Declaration and was approved by the Institutional Review Board of the Province of Jaen, Junta de Andalucia (GEN-VIH/0646-N-20 version 1 of 09/03/2020 and Protocol “Identificación de factores genéticos de Resistencia innata a la infección por VIH-1” of 26/07/2018). The participating centers and hospitals were: Complejo Hospitalario de Jaén (Jaén), Hospital Universitario Reina Sofía (Cordoba), S. Maria Annunziata Hospital (Florence) and Global Solutions for Infectious Diseases (San Francisco). All patients and healthy blood donors provided written informed consent to participate in this study.

## 3. Results

Sociodemographic and clinical variables of the subjects were previously described [45,46,47,48,49,50,51]. Two individuals in the HESN group were homozygous for CCR5 Δ32 and were excluded from subsequent analysis. Nine SNPs located in the CD46 locus were genotyped previously [40], and none of them, except rs2796265, showed a nominally significant association with HIV-1 status (*p* = 10^−3^) (Supplementary Table S1).

A strong deviation from Hardy–Weinberg equilibrium was observed for rs2796265 in IDU HIV-1 positive (*p* = 0.005) and sexual groups (*p* = 0.03). No departure from Hardy–Weinberg equilibrium was observed in the IDU (*p* = 0.3) or sexual (*p* = 0.4) HESN cohorts (Table 1).

The comparison of genotype frequencies revealed a remarkably differential distribution of the CD46 rs2796265 genotypes in HIV-1 positive patients compared to HESN subjects (Table 1). Thus, the frequency of homozygous individuals with the minor allele (CC) was significantly higher in IDU HESN subjects compared to IDU HIV-1 positive subjects (6% vs. 0.004%, *p* = 0.001). Minor allele homozygotes (CC vs. CT + TT) appear as a resistance factor for HIV-1 infection (OR = 0.07; *p* = 0.001; Table 1). A similar trend (although not statistically significant) was found when comparing the sexually exposed HESN with HIV-1 positive subjects (OR = 0.3; *p* = 0.06; Table 1). Combining all samples confirms the association with a lower risk of infection for the minor allele under a recessive model C/C vs. C/T + T/T; OR = 0.13, *p* = 0.0001 (Table 1).

**Figure 1:**
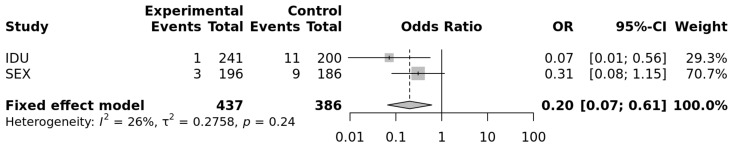
Forrest plot of meta-analysis of the sexual and IDU populations: OR and 95% Confidence interval and fixed-effect meta-analysis. *p* = 0.0044 for a recessive model of the protective allele (https://metagenyo.genyo.es/ (accessed on 13 June 2021)).

The results of the two populations at risk of parenteral and sexual HIV-1 transmission were combined through a fixed-effect meta-analysis and yielded a significant difference for the comparison including the genotypes CC vs. CT + TT (OR = 0.2; *p* = 0.0045; heterogeneity test *p* = 0.24). The evaluation of the CC vs. TT genotypes yielded a similar OR (0.19; *p* = 0.0044; heterogeneity *p* = 0.26; Figure 1), compatible with a recessive mode of expression of this protective factor concerning HIV-1 susceptibility.

**Figure 2:**
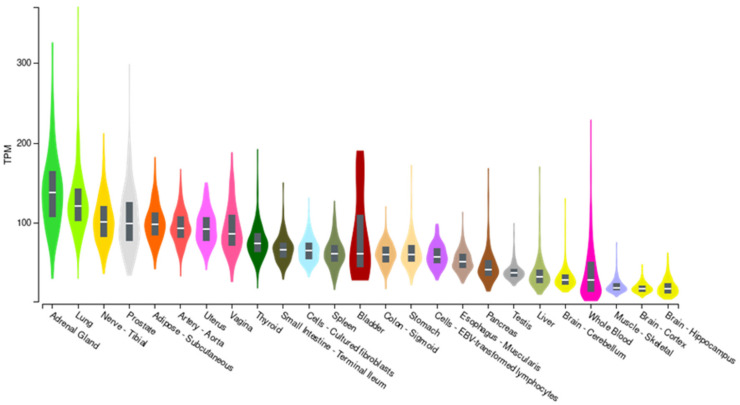
CD46 expression in different tissues (GTEX portal). TMP: Transcripts per million reads.

The role of the rs2796265 polymorphism in the expression of the CD46 gene was investigated through the GTEx portal (ReleaseV7; https://www.gtexportal.org (accessed on 19 June 2021)). CD46 mRNA is ubiquitously expressed in human tissues (Figure 2).

**Figure 3:**
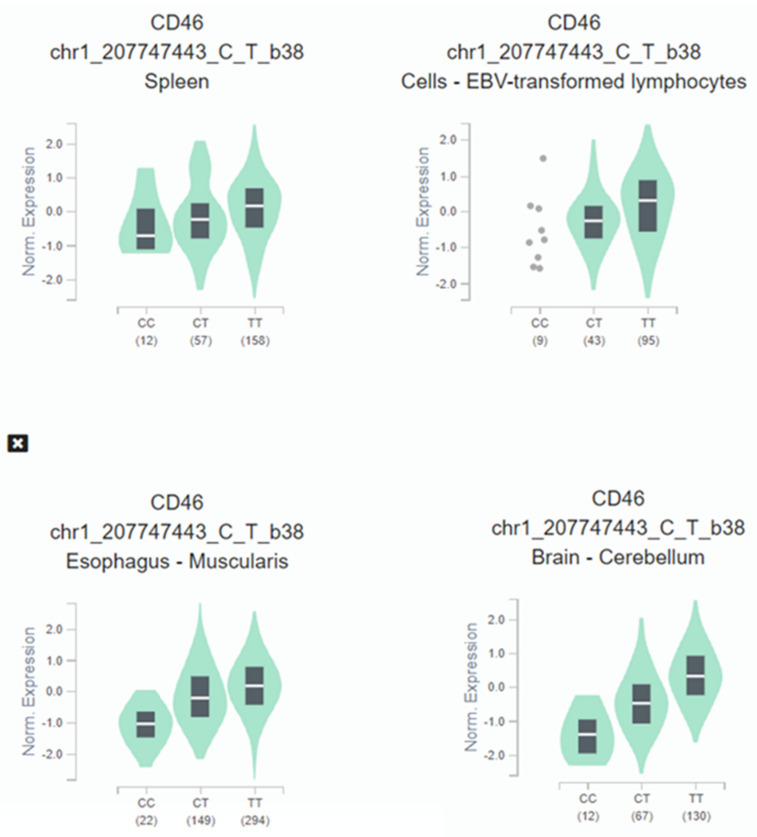
Illustrative violin plot where CD46 normalized expression is plotted according to the rs2796265 genotypes determined in cultured fibroblasts, spleen, brain, and immortalized B-lymphocytes according to GTEX portal. Protective genotype CC show significative lower levels of expression in several representative tissues: Esophagus (*p* = 5.9 × 10^−53^), brain (*p* = 1.7 × 10^−36^), spleen (*p* = 3.1 × 10^−7^), immortalized B-lymphocytes (*p* = 1.0 × 10^−6^). The values between parentheses represent the number of samples included in each genotype.

When expression-quantitative trait locus (eQTL) analysis was performed, we observed that the HIV-1 protective C allele correlated with lower CD46 expression across several tissues (Figure 3). CD46 expression in homozygotes C/C subjects is significantly decreased: oesophagus (*p* = 5.9 × 10^−53^), brain (*p* = 1.7 × 10^−36^), spleen (*p* = 3.1 × 10^−7^), and immortalized B-lymphocytes (*p* = 1.0 × 10^−6^).

**Figure 4:**
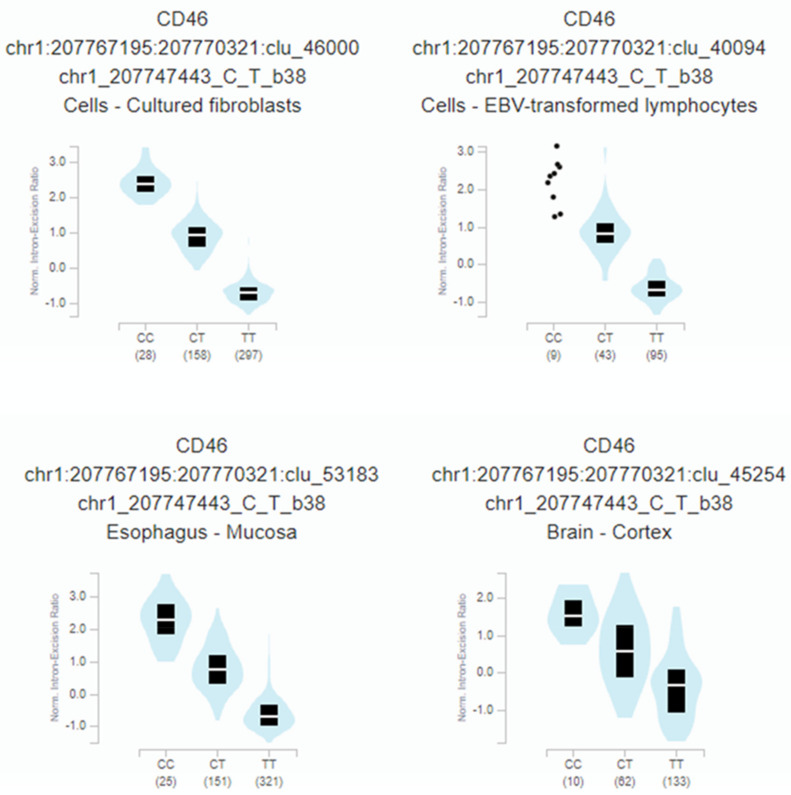
Relationship between the rs2796265 SNP genotypes and the normalized intron-excision ratio in four representative tissues: Fibroblast (*p* = 1.4 × 10^−259^), esophagus (*p* = 1.7 × 10^−188^), immortalized B-lymphocytes (*p* = 2.1 × 10^−57^), brain-cortex (*p*= 1.3 × 10^−25^). The splicing phenotype IDs are constructed by LeafCutter, and indicate the intron (chr:start:end) and cluster of connected components (clu_) the intron belongs to [44].

Furthermore, rs2796265 genotypes correlate with a highly significant CD46 mRNA splicing quantitative locus in several tissues: fibroblast (*p* = 1.4 × 10^−259^), oesophagus (*p* = 1.7 × 10^−188^), immortalized B-lymphocytes (*p* = 2.1 × 10^−57^), or brain-cortex (*p* = 1.3 × 10^−25^) (Figure 4).

## 4. Discussion

The function of CD46 in innate and adaptive immune responses is a matter of considerable interest as restriction of CD46-mediated signaling pathways is associated with innate immunodeficiency. Individuals with sporadic severe mutations in *CD46* cannot generate a Th1 response and develop recurrent severe infections [18]. In parallel, patients with mutations in Jagged-1, a CD46 ligand, suffer from Alagille Syndrome and recurrent viral infections due to deficient protective Th1 immunity [18]. Common *CD46* polymorphisms are also associated with immune-related phenotypes such as febrile seizures following virus vaccination [52], neutralizing antibody responses to measles vaccine [53], and blood monocyte counts [54]. Other unrelated phenotypes such as atypical hemolytic uremic syndrome, heart rate response to exercise and recovery, and blood pressure, preeclampsia, spontaneous pregnancy loss, systemic sclerosis, glomerulonephritis, and thrombotic thrombocytopenic purpura have been also associated with *CD46* gene variation [14], highlighting the pleiotropic function of this protein.

Our finding that the minor allele of rs2796265 is significantly underrepresented in HIV-1 infected individuals supports a role for *CD46* in the HESN phenotype. Moreover, the association with HIV-1 susceptibility represents a new phenotype related to the *CD46* gene. Although the finding does not reach statistical significance in individuals at risk through the sexual route, we observe the same trend as found in IDU. The smaller number of subjects in the sex HESN group may explain the lack of significant results in this population. The sexually exposed non-infected individuals have an a priori lower risk of HIV-1 compared to exposed intravenous drug users and some sex HESN could be misclassified.

The SNP rs2796265 is located in the promoter region of *CD46*, and the alleles are correlated to mRNA levels and splicing isoforms ratio supporting a role in the pathogenesis of HIV-1 infection, as previously suggested [10]. It is plausible that lower complement inhibitory activity of the isoforms expressed by the minor allele may lead the virus more susceptible to complement attack. Alternatively, the explanation for the association here reported may be related to the complex immune regulatory networks depending on CD46 signaling as Th1 response. The SNP rs2796265 belongs to a linkage disequilibrium block that includes several intronic SNPs. All of the minor alleles of this block (containing the HIV-resistance allele) have been found to influence at least two phenotypes. Minor allele homozygotes of rs2796265 showed about half the median neutralizing antibody titer against measles virus after vaccination compared with the major allele [53]. Paradoxically, the same individuals produce more IFNα in PBMC cultures after in vitro stimulation [53]. The correlation between the protective genotype and higher IFNα production in response to viral infection [53] could be an alternative explanation to the association here reported.

Four main *CD46* isoforms are resulting from alternative splicing in most human tissues. The extracellular portion of CD46 consists of four conserved short consensus repeats, an O-glycosylated area enriched in serines, threonines, and prolines (STP domain), a transmembrane segment, and a cytoplasmic tail (16 or 23 amino acids depending on differential splicing). *CD46* exon 7 and 8 are mutually exclusive [35], RNAseq data from previous studies [53] and those obtained by us, confirm that the haplotype carrying the SNP rs2796265 influences exon skipping and total *CD46* mRNA expression. Remarkably, exon 7 was found to be skipped in HIV-1 infected T cells, generating an alternative protein product lacking the extracellular STP region [35]. Moreover, lymphocytes and dendritic cells from HIV-1 infected patients show significative lower levels of extracellular CD46 expression, indicative that CD46 may influence the pathophysiology of the HIV-1 infection [9,35,36,38]. Future studies have to address the functional relevance of *CD46* genomic variation and its relationship with mRNA, protein expression, and its regulation by cytokines under the context of HIV-1 susceptibility and disease progression.

The main limitation of our research is the small population included in the meta-analysis and the possible bias due to the number of minor homozygotes, small random deviations can have consequences in the statistical significance. 

## 5. Conclusions

Our results suggest that *CD46* has a role in innate resistance to HIV-1 infection, which supports the importance of complement receptors and regulators in the pathogenesis of HIV infection. Further research and replication are needed to confirm this data and to explain how genetic variation in *CD46* influences innate immunity against HIV-1. If our results are confirmed, they may lead to a new potential target for pharmacological intervention in HIV-1 infection by modulation of CD46 signaling.

## 6. Patents

No patent pending related to this study.

## Figures and Tables

**Table 1:** Allelic and genotypic distribution in HIV-1 infected and HESN subjects.

IDU	Cohorts	Alleles ^a^	Genotypes ^a^		
		C	C/C	C/T	T/T
	HIV-1, n (%) *p* ^c^ = 0.005	83 (17.2)	1 (0.004)	81 (34)	159 (66)
	HESN, n (%) * p ^c^ = 0.3	83 (20.7)	11 (6.0)	65 (31)	118 (64)
	**Association test ^b^**	**C vs. T**	**C/C vs. T/T**	**C/C vs. C/T**	**C/C vs. C/T + T/T**
	OR (95% CI)	0.83 (0.58–1.2)	0.07 (0.009–0.5)	0.06 (0.008–0.5)	0.07 (0.009–0.5)
	*p*-value ^b^	0.18	0.001	0.001	0.001
**Sex**	**Cohorts**	**Alleles ^a^**		**Genotypes ^a^**	
		**C**	**C/C**	**C/T**	**T/T**
	HIV-1, n (%) *p* ^c^ = 0.031	71 (18)	3 (1.5)	65 (33.1)	128 (65.3)
	HESN, n (%) *p* ^c^ = 0.4	80 (22)	9 (4.8)	62 (3.3)	108 (61)
	**Association test ^b^**	**C vs. T**	**C/C vs. T/T**	**C/C vs. C/T**	**C/C vs. C/T + T/T**
	OR (95% CI)	0.81 (0.5–1.2)	0.32 (0.08–1.2)	0.3 (0.07–1.2)	0.31 (0.08–1.2)
	*p*-value ^b^	0.23	0.08	0.06	0.06
**IDU + Sex**	**Cohorts**	**Alleles ^a^**	**Genotypes ^a^**		
		**C**	**C/C**	**C/T**	**T/T**
	HIV-1, n (%) *p* ^c^ = *p* = 0.001	154 (17.8)	4 (0.01)	146 (33)	287 (65)
	HESN, n (%) *p* ^c^ = 0.3	163 (20.8)	20 (5.1)	123 (31)	243 (62)
	**Association test ^b^**	**C vs. T**	**C/C vs. T/T**	**C/C vs. C/T**	**C/C vs. C/T + T/T**
	OR (95% CI)	0.8 (0.6–1.0)	0.13 (0.03–0.04)	0.13 (0.03–0.04)	0.13 (0.03–0.04)
	*p*-value ^b^	0.07	0.0004	0.0003	0.0002

Notes: ^**a**^ Data are allele or genotype counts (%). **^b^** Fisher’s exact test. OR: Odds Ratio. CI: Confidence interval. ^c^ Test for deviation of Hardy–Weinberg equilibrium. * Individuals homozygous for *CCR5*∆32 and were excluded from this analysis.

## Data Availability

The data generated during the current study are available from the corresponding author on reasonable request.

## Supplementary Materials

The following are available online at https://www.mdpi.com/article/10.3390/cells10113094/s1, Table S1: Association of SNP in *CD46* locus with HIV-1 resistance in IDU.
