# Supplementary material for: CD46 Genetic Variability and HIV-1 Infection Susceptibility
Source: Cells. 2021 Nov 9;10(11):3094. doi: 10.3390/cells10113094 (PMC8622916; doi:10.3390/cells10113094)
Supplement: Supplementary file 1 [file cells-10-03094-s001.zip › cells-1418718 supplementary.pdf]

Table S1: Association of SNP in CD46 locus with HIV-1 resistance in IDU

SNP	Allele 1	Allele 2	Genotype counts HIV-1	Genotype counts HESN	Test	P
rs7541230	C	T	12,111,117	16,76,108	CC vs. CT+TT	0.19
rs2796266	A	G	21,115,104	20,84,96	AA vs. AG+GG	0.65
rs2796269	T	C	40,119,79	26,100,73	TT vs. Ct+tt	0.27
rs2796278	A	C	40,123,77	36,106,58	AA vs. AC+CC	0.71
rs2796267	G	A	32,103,106	26,83,91	GG vs. AG+AA	0.93
rs2724377	C	T	52,118,71	44,95,61	CC vs. CT+TT	0.91
rs2796265	C	T	1,81,159	11,61,128	CC vs. CT+TT	0.001
rs14374	C	T	0,17,223	0,11,189	TT vs. CT+CC	0.49
rs7545126	T	C	2,18,194	2,30,151	CC vs. CT+TT	0.01
